# Career Choices and Career Progression of Junior Doctors in Dermatology: Surveys of UK Medical Graduates

**DOI:** 10.1155/2018/2092039

**Published:** 2018-03-27

**Authors:** Atena Barat, Michael J. Goldacre, Trevor W. Lambert

**Affiliations:** UK Medical Careers Research Group, Unit of Health-Care Epidemiology, Nuffield Department of Population Health, University of Oxford, Old Road Campus, Oxford OX3 7LF, UK

## Abstract

**Objective:**

To report UK-trained doctors' career choices for dermatology, career destinations, and factors influencing career pathways.

**Methods:**

Multicohort multipurpose longitudinal surveys of UK-trained doctors who graduated between 1974 and 2015.

**Results:**

In all, 40,412 doctors (58% of graduates) responded in year 1, 31,466 (64%) in year 3, and 24,970 (67%) in year 5. One year after graduation, 1.7% of women and 0.6% of men made dermatology their first choice but by five years after graduation the respective figures were 1.0% and 0.7%. Compared to their predecessors, its popularity fell more substantially from years 1 to 5 among recent graduates (2005–15), particularly for women (from 2.1% in year 1 to 0.8% in year 5) compared with a fall from 0.8% to 0.5% among men. The most important factor influencing dermatology choice was “hours/working conditions”: in year one, 69% regarded this as important compared with 31% of those choosing other hospital physician specialties. Only 18% of respondents who chose dermatology at year 1 eventually worked in it; however, almost all practising dermatologists (94%), 10 years after qualifying, had made their future career decision by year 5.

**Conclusion:**

Dermatology is popular among female UK graduates. Most dermatologists made their career decision late but decisively.

## 1. Introduction

Dermatology is a highly competitive specialty to enter both globally and within the UK [1–3]. In the UK, all aspiring dermatologists begin their training by becoming fully registered with the General Medical Council (GMC). This is achieved by completing a two-year Foundation Programme which follows their medical degree and is common to all specialties. Trainees then spend two to three years in one of three core training programmes: Core Medical Training (CMT), which is the first stage of postgraduate training for many physician specialties; Acute Care Common Stem (ACCS) training, which is a training pathway parallel to CMT for those particularly interested in acute specialties; or Paediatrics. All intending dermatologists must attain, at the end of that period of training, membership of the Royal College of Physicians (MRCP) or membership of the Royal College of Paediatrics and Child Health (MRCPCH) before commencing specialty training in dermatology which takes at least four years. Finally, successful trainees are awarded their certificate of completion of training (CCT) and can apply for consultant dermatologist positions [4, 5]. In 2017, dermatology recorded the highest level of competition for training posts among all specialties in the UK, with 5.58 applicants for each available post [3].

Dermatology has some characteristics that make it appealing for medical graduates. It is considered a rewarding specialty due to recent advances in medicine which have rendered chronic diseases curable or controllable [5, 6]. Dermatologists interact with a variety of patients from all ages but they are rarely life threateningly unwell [5]. Since dermatology is mainly an outpatient-based specialty with a relatively low on-call commitment, flexible training is available and many of its consultants work less than full time [7, 8]. The controllable lifestyle features, including control of working hours, of dermatology make it popular among female doctors [2, 9, 10]: in the UK in 2016, 57% of consultants and 75% of higher specialty trainees in dermatology were women [11].

Dermatological conditions are extremely prevalent, accounting for around 13 million patients annually presenting to GPs in England and Wales; and nearly 6.1% (0.8 m) are referred for specialist consultation. In 2005, approximately 4000 deaths in the UK were ascribed to skin disease [12]. In the UK, multidisciplinary teams led by consultants provide specialist care services. The team may include specialist registrars (StR) in Dermatology, Staff and Associate Specialist (SAS) doctors, specialist dermatology nurses, general practitioners with a special interest (GPwSI) in dermatology, and specialist trainees [4, 12]. In 2016, 538 consultant and 259 medical registrars, in terms of full-time equivalents, were working in dermatology in England [13]. Ideally, 1.6 consultants per 100,000 population are recommended by the Royal College of Physicians [14], indicating a shortage of approximately 100 dermatology consultant posts [15], with particular shortages in rural or remote areas [16]; however, no region in the UK has sufficient dermatology consultants [14]. The reported shortfall and uneven distribution of dermatology professionals has resulted in unmet patient needs [16].

The distribution of physicians in different specialties across a country is affected by the career choices of medical graduates [2]. Thus, understanding their preferences for a career specialty can help to improve the educational programmes, plan for a balanced distribution of workforce, and evolve the health system to better suit the needs of specialties and the choices of doctors [17]. The goal of this paper is to report trends in choosing dermatology as a career over various cohorts, to identify factors which principally motivate medical graduates choosing dermatology, and to compare their early career intention with their eventual destination. The information should be helpful to workforce planners, providers of care, and educational commissioners in supporting the provision of an effective and sustainable health system. It should also help medical graduates to understand important issues related to dermatology careers.

## 2. Methods

### 2.1. Data Collection

We analysed a large national database generated during the multicohort surveys conducted by the UK Medical Careers Research Group. Detailed information about study design, methods, and participants has been reported previously [18]. Briefly, the target population was all UK-trained doctors, registered by the General Medical Council (GMC), since 1974, in particular years of graduation. Participants have been followed up with questionnaire surveys at the first, third, and fifth years after graduation and longer intervals thereafter. To enhance the reliability of the study and increase the response rate [19], up to four follow-up reminders were sent to those who were initially nonrespondents.

Our data related to 15 cohorts: the UK medical graduates of 1974, 1977, 1980, 1983, 1993, 1996, 1999, 2000, 2002, 2005, 2008, 2009, 2011, 2012, and 2015. The analysis focused on three main concepts: early career preferences, influencing factors on career choices, and eventual career destinations. Information on the first two measures was obtained from 15 cohorts (1974–2015), 12 cohorts (1974–2008 and 2012), and 10 cohorts (1974–1980 and 1993–2008) at the first, third, and fifth year after qualification, respectively. Information on the last measure, career destination, was obtained from 5 cohorts (1993–2002) at 10 years after graduation.

### 2.2. Research Instrument

A self-administered, postal or electronic, questionnaire containing sections on demographic information, career choices and plans, and employment history was completed by each respondent. The content and construct of the questionnaire were checked and revised over many years [20].

The questionnaire asked participants about their specialty preferences using the question “What is your choice of long-term career specialty?,” enabling up to three specialty choices to be specified in order of preference. The questionnaire also asked about certainty of choices. Participants could rate their level of certainty about their career choice as “definite,” “probable,” or “uncertain.” A three-point scale covering “not at all,” “a little,” and “a great deal” was used to explore participants' views regarding each of 13 influencing factors on career choices.

### 2.3. Data Analysis

To increase statistical power, we combined the individual cohort data into three groups: graduates of 1974–1983, 1993–2002, and 2005–2015. This classification corresponds to pivotal periods when changes happened in medical education in the UK.

In analysing specialty choices, we used the term “tied choices” to refer to choices of equal priority for a respondent. Similarly, “untied choices” refers to a solo choice that a participant explicitly named it as his/her preferred specialty. Moreover, hospital medical specialties other than dermatology, whose training is managed by the Joint Royal Colleges of Physicians Training Board (JRCPTB), were grouped together and named in this paper as “other hospital physician specialties” as a comparative group to compare with dermatologists. This group included the following: general medicine, cardiology, endocrinology, geriatrics, nephrology, neurology, chest medicine, rheumatology/rehabilitation, genitourinary medicine, gastroenterology, vascular medicine, tropical medicine, clinical pharmacology, infectious diseases, and occupational medicine. These trainees complete a similar core medical training (CMT) stage prior to their specialty training programme as dermatologists, and like dermatologists their working environment is mainly in hospital rather than in the community [21].

The statistical analyses were carried out with IBM SPSS Statistics for Windows, V22 [22], and Microsoft Excel (2010). Heterogeneity of cohort group was examined through the *χ*^2^ statistic test. Bivariate cross-tabulation and Chi-square test with the appropriate number of degrees of freedom were applied for comparison of proportions and the Mantel–Haenszel linear-by-linear *χ*^2^ test was performed to identify linear trends over cohorts. The corresponding 95% confidence interval was calculated and statistical significance inferred at a 2-tailed probability value < 0.05. All analysis was reported stratified by sex and cohort group and all results are also presented as sex-combined.

## 3. Results

### 3.1. Response Rate

In the first year after graduation, we contacted 69,670 registered doctors covering all 15 cohorts: 40,412 (58.0%) replied. The questionnaire was sent to 48,899 subjects in year 3 covering all 12 cohorts and 31,466 (64.3%) responded. For five years after qualification, covering 10 cohorts, 24,970 from a possible 37,424 doctors (66.7%) replied.

### 3.2. Early Career Choices for Dermatology: One, Three, and Five Years after Graduation

In total, the percentage of doctors who specified dermatology as their first choice showed a gradual fall from 1.2% in year 1 to 0.9% in year 5 (Table 1). Testing for trend across the three cohort groups defined by the graduates of 1974–1983, 1993–2002, and 2005–2015, in each row of Table 1, no trend was found for years 3 and 5, but a linear upward trend was found for year 1 (*χ*^2^ = 41.8, df = 1).

Examination of individual groups of cohorts (Table 1) showed no evident trend in the popularity of dermatology as a first choice of long-term career, moving from year 1 to year 5, for the two earlier groups (1974–83 and 1993–2002), but a downward trend for the recent group (2005–15), with only 0.7% of the cohorts of 2005 and 2008 choosing dermatology in year 5 compared to 1.4% (92/6430 from Table 2) of those cohorts in year 1.

Results for individual cohorts are shown in Table 2.

### 3.3. Certainty of Career Choice

Doctors who chose dermatology were surer of their choice than doctors who chose other hospital specialties (Table 3). Their level of certainty rose from 18% in year 1 to 72.6% in year 5. Similar increases in certainty applied to both genders. Male doctors regardless of their chosen specialty were more certain of their choice than female doctors.

### 3.4. Choices by Gender

**Figure 1:**
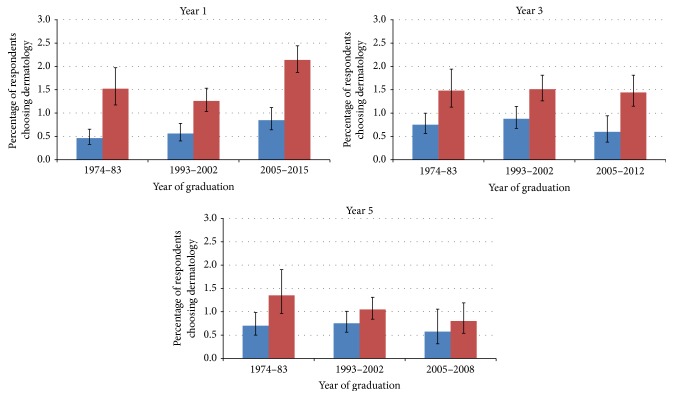
Percentages of men (blue) and women (red) doctors who chose dermatology as a first choice of eventual career one, three, and five years after graduation.

Over all cohort groups and all years of graduation, higher percentages of women than men chose dermatology as their first choice of future career (Table 1, Figure 1). Nevertheless, its popularity for both genders varied across cohorts and survey years. Comparing year 1 with year 5 across all cohorts, choices for dermatology for men remained roughly constant at 0.6% and 0.7%, respectively, while for women choices fell from 1.7% to 1.0%.

Comparing cohort groups in year 1, we identified an upward trend in choosing dermatology from 1974 to 2015 for both genders but the statistical evidence for the trend was weaker for men (men: *χ*^2^ = 8.07, *p* = 0.018; women: *χ*^2^ = 21.06, *p* < 0.001, any first choice). Gender differences continued in years 3 and 5 but showed a downward trend across cohorts in the percentages of both male and female doctors choosing dermatology as a first choice, with nonsignificant results. Generally, the difference between men and women who chose dermatology increased from 1974 to 2015 in years 1 and 3 but narrowed in year 5.

Gender variation was reduced at all three cohort groups, moving from years 1 to 5. The most recent cohort group (2005–15) showed the largest gender gap in year 1 (1.29%) and the smallest in year 5 (0.23%). Among women, choices for dermatology fell from 2.1% to 0.8%, while among men the fall was only from 0.8% to 0.5%, comparing year 1 with year 5.

**Figure 2:**
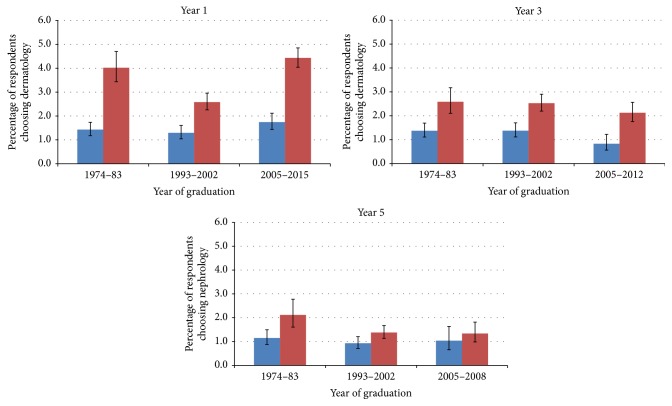
Percentages of men (blue) and women (red) doctors who chose dermatology as a first, second, or third choice of eventual career one, three, and five years after graduation.

The gender balance in choices for dermatology was similar whether for the specialty as first choice or as any choice (Figure 2).

### 3.5. Factors Influencing Choice for Dermatology

Table 4 shows the percentages of dermatologists and of those working in other hospital physician specialties who specified, for each factor, whether it influenced their career choice a great deal.

Hours and working conditions were the most important factor for doctors who chose dermatology, the importance increasing as time passed from qualification. The factor scoring the second highest percentage for aspiring dermatologists was enthusiasm and commitment. Other important factors for aspiring dermatologists were domestic circumstances, self-appraisal, and student experience of the subject. In contrast, financial circumstances while training, inclinations before medical school, and advice from others were unimportant.

Compared with doctors who chose other hospital physician specialties, dermatologists scored consistently higher on the importance of hours and working conditions, domestic circumstances, future financial prospects, and career and promotion prospects, but less highly on experience of jobs so far.

### 3.6. Comparing Early Choice with Eventual Destination

#### 3.6.1. Looking Forward to Eventual Destination

Early career choices for dermatology in years 1, 3, and 5 were matched with four career destination groups in year 10 after qualification (Table 5). Only 19% of respondents who chose dermatology in year 1 were working as dermatologists in year 10. This percentage increased substantially when year 3 and 5 choices were considered, reaching 44% and 85%, respectively. More than half of the respondents who specified dermatology as their first choice in year 1 did not become hospital physicians and more than a quarter of them were in general practice in year 10 including 32% of the women who had chosen dermatology (Table 5). Only very small numbers of those who chose dermatology in year 5 were not working in the specialty at year 10.

#### 3.6.2. Looking Backwards to Early Choices

Looking backwards, 30% of practising dermatologists in year 10 had considered dermatology as a future career in year 1, whether as first, second, or third choice (Table 6). Looking back from year 10 destinations to year 3 and 5 choices in the same way indicated much higher percentages which reached 70.3% and 94%, respectively.

## 4. Discussion

### 4.1. Main Findings

Considering choices for dermatology one year after graduation, there was an upward trend across the year of graduation cohorts. However, interest fell in years three and five, and the percentage of doctors in their fifth postqualification year who chose dermatology was much lower, particularly among women and particularly in the most recent year of graduation cohorts. Research into the causes of this loss of initial interest is suggested. Nevertheless, throughout this study dermatology was more popular among female than male doctors, though this gender difference gradually diminished as time passed from graduation. A third of women who chose dermatology one year after graduation were later working in general practice.

Across all cohorts, three life style and income related factors (i.e., “hours/working conditions,” “domestic situation,” and “future financial prospects”) were more important to intending dermatologists than doctors opting for other hospital medical specialties. The first two factors were more important for female than male doctors while the latter was more influential for male respondents. Aspiring dermatologists scored lower on “experience of jobs so far” than those choosing other hospital specialties. This may reflect low exposure to dermatology in medical school or in the early years after graduation.

Dermatologists were late decision-makers regarding their future career. Over two-thirds of practising dermatologists had not chosen dermatology in their first year after graduation, whereas, in year 5, 94% of them had given dermatology as their first choice. Less than one-fifth of intending dermatologists in year 1 eventually became dermatologists; but the corresponding figure exceeded four-fifths in year 5. Confidence in dermatology choices increased with time for both female and male participants. Considering our data on influencing factors, their relatively late but assured decisions to commit to a career in dermatology could be attributed in part to the field experiences or self-awareness which had been built up over time.

### 4.2. Comparison with Other Research

In this longitudinal study, recent medical graduates were gradually losing their interest in pursuing dermatology as their future career between years 1 and 5. This downward trend was not unique for dermatology and also observed in other specialties such as nephrology [23].

The gender differences in specialty preference in our results were highlighted in studies from other countries, for example, Japan [24] and the US [10]. The reported high rate of women in dermatology in our study corresponds with previous research. For instance, in Japan, dermatology was mentioned as a popular career choice among female physicians [24]. A study of over 11,000 medical students from 11 countries in Latin America indicated female gender was associated with the choice of dermatology [25].

Additionally, life style and income related factors are valued as determinants of motivation for choosing dermatology by our respondents. The “work–life balance” benefit of dermatology was emphasized by not only the UK-trained doctors, but also medical graduates from both developed and underdeveloped countries [2]. In a study of fourth-year medical students in the US, dermatology was rated the highest scoring specialty for lifestyle. Controllable lifestyle in the US study comprised four items: predictable working hours, having extra time for activities outside of work, leisure, and being with family [10]. Further, a cross-sectional study on 200 Saudi medical students found that the majority of respondents were attracted to dermatology by having roots in life-work balance as well as financial and occupational satisfaction, including how dermatologists lead a satisfying family life, the appeal of being a dermatologist, opportunities for researches, and the reliance on clinical skills for diagnosis. It is notable that 66% of intending dermatologists in that study were females.

Although, in recent years, specialties with controllable lifestyle and work–life balance are highlighted by both male and female doctors [9, 26], their importance is higher for many women [27] which may be due to women's traditional roles in supporting family and competing demands for the time of family and career [28]. Considering dermatology as a career with controllable lifestyle [29] and the tendency of females towards professions compatible with their family life [2], the higher proportion of women in dermatology is understandable. In this respect, for many doctors dermatology will compare well with other specialties such as surgery [30].

Late career decisions among aspiring dermatologists were also reported in other research among those intending other hospital specialties such as cardiology [31]. As time passes from qualification, medical graduates will gain a better assessment of their abilities and a realistic vision of expected career roles through their first-hand experiences and contact with more experienced and informed colleagues [32]. Literature suggests that later preferences encompass factors beyond job values, work–family, and encouragement and mentoring from others [33].

### 4.3. Implications

The greater influx of female than male doctors in dermatology field, particularly in recent years, may raise concern regarding workforce gender imbalance. Although, in some countries, training and practice in dermatology are combined with venereology [34], in the United Kingdom, genitourinary medicine is a separate specialty [5]. Hence concerns which may be expressed, for example, by a male patient hesitating to discuss their complaint with a female dermatologist would not normally apply in the UK. However, an increasingly female gender balance in the specialty may lead to an increase in part-time working and a consequent reduction in capacity. Further research is needed to elucidate the working status of dermatologists and provide a comprehensive picture of the workforce in this specialty.

Late decisions to commit to a future career are mainly related to applicants' lack of information about their desired specialty, which can be tackled through preparing both clinical and research mentorship programmes [35], offering specialty-specific electives during medical schools [36], and providing career-support services [36]. Late career choice is difficult for applicants and risks wasting societies' resources and talents. Thus, it is crucial to recognise the aptitudes of medical students as early as possible and lead them towards a proper career path tailored to their needs and talents. In doing so, constant and concerted support from all stakeholders particularly educational providers and workforce planners is required.

### 4.4. Strengths and Limitations

This, the longest multicohort national study, provides an insight into the views of UK medical graduates on dermatology choices and provides an initial point of investigation to start more specific evaluation of their training and experiences. The relatively large sample size and the prospective design over 10 years enable comparison of choices, career outcomes, and motivations. However, some limitations exist. We do not include physicians working in the UK who graduated abroad and, as in any survey work, there is a possibility of responder bias.

## 5. Conclusions

In summary, the popularity of dermatology was evidently higher among female than male participants across all cohorts. Intending dermatologists significantly valued life style and income related factors higher than those intending to work in other hospital specialties. Furthermore, dermatology choices were relatively stable in the later stages in the career course and changes regarding career decisions tend to occur in the first few years after medical school. Thought should be given to future administrative strategies that can improve the work–life balance in other specialties and support medical students regarding their career choice.

## Figures and Tables

**Table 1:** Trends in early first choices for eventual career in dermatology: percentages of doctors who specified dermatology at years 1, 3, and 5 after graduation.

Year after graduation	Cohorts (years of graduation)				Statistical tests	
	1974–83 *N* (%)	1993–2002 *N* (%)	2005–15 *N* (%)	All cohorts *N* (%)	Linear trend (*χ*^2^1, *p*)	Heterogeneity (*χ*^2^2, *p*)
*Men and women*						
Year 1	88 (0.8)	133 (0.9)	263 (1.7)	484 (1.2)	41.8, <0.001	48.7, <0.001
Year 3	98 (1.0)	169 (1.2)	90 (1.1)	357 (1.1)	0.64, 0.42	2.41, 0.29
Year 5	65 (0.9)	121 (0.9)	34 (0.7)	220 (0.9)	1.06, 0.3	1.76, 0.41
*Men*						
Year 1	32 (0.5)	35 (0.6)	49 (0.8)	116 (0.6)	7.4, 0.006	8.07, 0.018
Year 3	47 (0.8)	54 (0.9)	18 (0.6)	119 (0.8)	0.23, 0.62	2.11, 0.34
Year 5	33 (0.7)	44 (0.8)	10 (0.6)	87 (0.7)	0.09, 0.75	0.61, 0.73
*Women*						
Year 1	56 (1.5)	98 (1.3)	214 (2.1)	368 (1.7)	12.3, <0.001	21.06, <0.001
Year 3	51 (1.5)	115 (1.5)	72 (1.4)	238 (1.5)	0.03, 0.85	0.09, 0.95
Year 5	32 (1.4)	77 (1.1)	24 (0.8)	133 (1.0)	3.85, 0.05	3.87, 0.14

**Table 2:** Choices for dermatology in individual cohorts: percentages and numbers of respondents.

Graduation year	Year 1 % (*N*)	Year 3 % (*N*)	Year 5 % (*N*)	Year 1 % (*N*)	Year 3 % (*N*)	Year 5 % (*N*)
	Dermatology as any first choice			Dermatology as any choice		
1974	0.9 (18/1940)	1.2 (18/1486)	1.0 (18/1755)	2.1 (41/1940)	2.0 (29/1486)	1.4 (25/1755)
1977	0.8 (20/2637)	1.3 (31/2336)	1.2 (30/2602)	2.5 (66/2637)	2.5 (59/2336)	2.0 (52/2602)
1980	1.0 (29/2853)	1.0 (28/2843)	0.6 (17/2716)	2.9 (84/2853)	1.9 (54/2843)	1.0 (27/2716)
1983	0.7 (21/3166)	0.7 (21/3037)	0.0	1.8 (56/3166)	1.1 (33/3037)	0.0
1993	1.0 (26/2621)	1.0 (27/2777)	0.9 (24/2729)	1.9 (49/2621)	1.4 (39/2777)	1.1 (31/2729)
1996	0.7 (21/2926)	0.7 (20/2721)	0.7 (28/2521)	1.7 (51/2926)	1.4 (39/2721)	1.0 (24/2521)
1999	0.8 (21/2727)	1.5 (39/2549)	1.4 (38/2661)	1.7 (45/2727)	2.3 (59/2549)	1.8 (47/2661)
2000	1.2 (35/2978)	1.7 (51/2968)	1.0 (27/2703)	2.0 (59/2978)	2.7 (80/2968)	1.4 (38/2703)
2002	1.1 (30/2778)	1.2 (32/2748)	0.5 (14/2552)	2.8 (78/2778)	2.2 (60/2748)	0.6 (15/2552)
2005	1.2 (36/3128)	0.7 (20/2710)	0.6 (13/2362)	3.1 (96/3128)	1.3 (34/2710)	1.0 (24/2362)
2008	1.7 (56/3302)	1.2 (39/3228)	0.9 (21/2369)	3.8 (124/3302)	1.8 (58/3228)	1.4 (34/2369)
2009	1.7 (51/2917)	0.0	0.0	3.8 (110/2917)	0.0	0.0
2011	1.6 (16/1001)	0.0	0.0	3.1 (31/1001)	0.0	0.0
2012	1.9 (45/2398)	1.5 (31/2063)	0.0	3.6 (86/2398)	1.9 (39/2063)	0.0
2015	1.9 (59/3040)	0.0	0.0	3.2 (97/3040)	0.0	0.0

**Table 3:** Comparison of the firmness of choice between doctors who expressed a first preference for each specialty group at 1, 3, and 5 years after graduation.

Gender	Level of certainty of career choice	Year 1		Year 3		Year 5	
		Dermatology *N* (%)	Other hospital physician specialties *N* (%)	Dermatology *N* (%)	Other hospital physician specialties *N* (%)	Dermatology *N* (%)	Other hospital physician specialties *N* (%)
Male	Definite	22 (19.6)	664 (15.0)	37 (31.1)	707 (28.1)	68 (79.1)	1101 (61.1)
	Probable	54 (48.2)	2356 (53.2)	62 (52.1)	1276 (50.7)	14 (16.3)	570 (31.6)
	Uncertain	36 (32.1)	1407 (31.8)	20 (16.8)	533 (21.2)	4 (4.7)	131 (7.3)
	Total	112 (100.0)	4427 (100.0)	119 (100.0)	2516 (100.0)	86 (100.0)	1802 (100.0)

Female	Definite	64 (17.5)	522 (10.7)	62 (26.4)	507 (22.7)	91 (68.4)	968 (59.6)
	Probable	195 (53.4)	2294 (47.2)	104 (44.3)	1054 (47.3)	34 (25.6)	515 (31.7)
	Uncertain	106 (29.0)	2043 (42.0)	69 (29.4)	668 (30.0)	8 (6.0)	140 (8.6)
	Total	365 (100.0)	4859 (100.0)	235 (100.0)	2229 (100.0)	133 (100.0)	1623 (100.0)

All (men & women)	Definite	86 (18.0)	1186 (12.8)	99 (28.0)	1214 (25.6)	159 (72.6)	2069 (60.4)
	Probable	249 (52.2)	4650 (50.1)	166 (46.9)	2330 (49.1)	48 (21.9)	1085 (31.7)
	Uncertain	142 (29.8)	3450 (37.2)	89 (25.1)	1201 (25.3)	12 (5.5)	271 (7.9)
	Total	477 (100.0)	9286 (100.0)	354 (100.0)	4745 (100.0)	219 (100.0)	3425 (100.0)

For details of specialties included as “other hospital physician” specialties, see Methods.

**Table 4:** Numbers and percentages of doctors who specified each factor affecting their career choice “a great deal.”

Factor	Year 1		Year 3		Year 5	
	Dermatology % (*N*)	Other hospital physician specialty % (*N*)	Dermatology % (*N*)	Other hospital physician specialty % (*N*)	Dermatology % (*N*)	Other hospital physician specialty % (*N*)
Hours/working conditions	69% (211^*∗∗∗*^)	31% (1793)	75% (105^*∗∗∗*^)	35% (769)	82% (115^*∗∗∗*^)	38% (981)
Domestic circumstances	54% (195^*∗∗∗*^)	23% (1605)	45% (106^*∗∗∗*^)	22% (731)	44% (90^*∗∗∗*^)	27% (884)
Future financial prospects	25% (59^*∗∗∗*^)	9% (410)	25% (34^*∗∗∗*^)	7% (166)	17% (24^*∗∗∗*^)	7% (193)
Career and promotion prospects	29% (80^*∗∗*^)	22% (1250)	48% (96^*∗∗∗*^)	27% (785)	36% (75^*∗∗∗*^)	25% (834)
Experience of jobs so far	34% (79^*∗∗∗*^)	54% (2792)	41% (97^*∗∗∗*^)	59% (1989)	51% (106^*∗∗∗*^)	66% (2197)
Self-appraisal	43% (120^*∗*^)	49% (2939)	51% (122)	55% (1852)	54% (112)	57% (1894)
Student experience of subject	38% (131)	40% (2620)	33% (71^*∗*^)	26% (814)	31% (59^*∗∗*^)	23% (718)
A particular teacher/department	33% (89)	29% (1680)	30% (59^*∗*^)	37% (987)	30% (49^*∗*^)	39% (1045)
Inclinations before medical school	5% (14^*∗*^)	9% (531)	6% (14)	8% (245)	7% (14)	7% (211)
Enthusiasm/commitment	54% (164)	60% (3492)	65% (90)	70% (1550)	80% (113)	80% (2094)
Advice from others	17% (48)	17% (974)	21% (42)	20% (571)	17% (36)	16% (548)
Other reasons	11% (11)	13% (260)	16% (17)	18% (260)	26% (19)	24% (273)
Financial circumstances while training	6% (3)	3% (31)	5% (6)	5% (82)	3% (3)	6% (71)

*∗* indicates  *p* < 0.05;  *∗∗* indicates  *p* < 0.01;  *∗∗∗* indicates  *p* < 0.001, comparing dermatology with other hospital physician specialties, within each year, for each factor. For details of specialties included as “other hospital physician” specialties, see Methods.

**Table 5:** Numbers and percentages of medical graduates whose original choice was dermatology and eventually practised at four different destinations in year 10 (looking forwards).

	Four final destinations after 10 years				
	Dermatology % (*N*)	Other hospital physician specialties % (*N*)	Other clinical specialties % (*N*)	GP % (*N*)	Total % (*N*)
*Year 1 first choice*					
Male choosing dermatology	17% (5)	31% (9)	42% (12)	10% (3)	100% (29)
Female choosing dermatology	20% (15)	16% (12)	32% (24)	32% (24)	100% (75)
Total choosing dermatology	19% (20)	20% (21)	35% (36)	26% (27)	100% (104)
*Year 3 first choice*					
Male choosing dermatology	48% (21)	18% (8)	18% (8)	16% (7)	100% (44)
Female choosing dermatology	41% (36)	14% (12)	24% (21)	21% (18)	100% (87)
Total choosing dermatology	44% (57)	15% (20)	22% (29)	19% (25)	100% (131)
*Year 5 first choice*					
Male choosing dermatology	89% (33)	5% (2)	3% (1)	3% (1)	100% (37)
Female choosing dermatology	82% (46)	7% (4)	9% (5)	2% (1)	100% (56)
Total choosing dermatology	85% (79)	6% (6)	6% (6)	3% (2)	100% (93)

For details of specialties included as “other hospital physician” specialties, see Methods. “Other clinical specialties” includes the following: emergency medicine, anaesthesia, clinical oncology, surgery, paediatrics, pathology, psychiatry, and radiology.

**Table 6:** Numbers and percentages of practicing dermatologists in year 10, based on their original any first career choices in years 1, 3, and 5 (looking backwards).

Career choices	Male % (*N*)	Female % (*N*)	Total % (*N*)
*Year 1*			
Dermatology as untied first choice	15% (5)	22% (11)	19% (16)
Dermatology as tied 1st choice	0% (0)	8% (4)	5% (4)
Dermatology as 2nd or 3rd choice	9% (3)	4% (2)	6% (5)
Other hospital physician specialties	58% (19)	43% (21)	49% (40)
Others	18% (6)	22% (11)	21% (17)
Total	100% (33)	100% (49)	100% (82)
*Year 3*			
Dermatology as untied first choice	62% (21)	47% (27)	53% (48)
Dermatology as tied 1st choice	0% (0)	16% (9)	10% (9)
Dermatology as 2nd or 3rd choice	0% (0)	12% (7)	8% (7)
Other hospital physician specialties	15% (5)	10% (6)	12% (11)
Others	23% (8)	14% (8)	18% (16)
Total	100% (34)	100% (57)	100% (91)
*Year 5*			
Dermatology as untied first choice	100% (33)	88% (45)	93% (78)
Dermatology as tied 1st choice	0% (0)	2% (1)	1% (1)
Dermatology as 2nd or 3rd choice	0% (0)	0% (0)	0% (0)
Other hospital physician specialties	0% (0)	0% (0)	0% (0)
Others	0% (0)	10% (5)	6% (5)
Total	100% (33)	100% (51)	100% (84)

For details of specialties included as “other hospital physician” specialties, see Methods.
